# Highly Vertically Oriented Graphene Microstrip Pads With Ultrahigh Through‐Plane Thermal Conductivity and Ultralow Compressive Modulus for Efficient Heat Dissipation

**DOI:** 10.1002/advs.75359

**Published:** 2026-04-17

**Authors:** Xu Ran, Yaru Wang, Sijia Wu, Hejun Wang, Junhao Shen, Litao Sun, Xing Wu, Hengchang Bi

**Affiliations:** ^1^ In Situ Devices Center School of Integrated Circuits East China Normal University Shanghai P. R. China; ^2^ SEU‐FEI Nano‐Pico Center Key Laboratory of MEMS of Ministry of Education Collaborative Innovation Center for Micro/Nano Fabrication Device and System Southeast University Nanjing P. R. China

**Keywords:** compressive modulus, graphene microstrips, thermal conductivity, thermal interface materials, vertical orientation

## Abstract

The increasing heat generation in microelectronic devices demands efficient thermal interface materials (TIMs) to prevent overheating. However, the persistent trade‐off between high through‐plane thermal conductivity and excellent compliance remains a fundamental barrier for TIMs. Herein, we prepare a vertically oriented graphene microstrip pad (GMP) with both ultrahigh thermal conductivity and ultralow compressive modulus by aligning graphene microstrips vertically and encapsulating them in silicone rubber. The graphene microstrips align vertically with excellent orientation, forming continuous pathways for heat conduction. By optimizing the graphene content to 60 wt.% (GMP60), we achieve an ultrahigh through‐plane thermal conductivity of 565.92 W m^−^^1^ K^−^^1^. Benefiting from the low stiffness of graphene microstrips, GMP60 exhibits an ultralow compressive modulus of 115.16 kPa and a total thermal resistance as low as 0.028 in^2^ K W^−^^1^, thereby overcoming the traditional thermal‐mechanical trade‐off. Practical cooling tests confirm the superior performance of GMP60. It lowers the central processing unit (CPU) core temperature by 12°C under full‐load operation and reduces the power chip temperature by 18.2°C. This work establishes a new paradigm for overcoming thermal‐mechanical mismatches in next‐generation electronic thermal management.

## Graphical abstract

Vertically aligned graphene microstrip pads (GMPs) break the traditional thermomechanical trade‐off in thermal interface materials. It simultaneously achieves an ultrahigh through‐plane thermal conductivity of 565.92 W m^−^^1^ K^−^^1^ and an ultralow compressive modulus below 115.16 kPa. This novel structure enables superior cooling performance and long‐term reliability for advanced electronics thermal management.

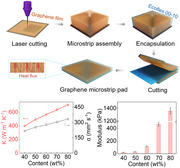

## 1 Introduction

Driven by emerging applications such as 5G communications, artificial intelligence, and high‐performance computing, modern electronic devices are moving toward miniaturization and integration [1, 2, 3, 4, 5, 6, 7, 8]. The rapid increase in power density and the more limited space in electronic devices lead to substantially higher device temperatures, compromising the reliability, operational stability, and lifespan of the electronic components [9, 10, 11]. For example, for GaN radio frequency devices, every 10°C increase in junction temperature reduces the device's average lifespan to half of its original value [12]. TIMs play a vital role by filling microscopic gaps between the device and heat sink, eliminating insulating air layers. Ideal TIMs must exhibit high thermal conductivity, low thermal resistance, and good compliance for efficient heat dissipation. Therefore, developing advanced TIMs that combine high thermal conductivity with low compressive modulus is urgently needed to overcome the thermal management bottleneck in high‐power‐density electronic devices.

To address increasingly severe thermal challenges, developing TIMs with ultrahigh through‐plane thermal conductivity has become a key research focus [13, 14, 15, 16, 17, 18]. Conventional TIMs typically combine polymer matrices with high‐loading fillers such as metallic particles (Cu, Ag) or ceramic powders (Al_2_O_3_, AlN, BN) [19, 20, 21, 22]. While metallic fillers provide high intrinsic thermal conductivity (e.g., Cu: 401 W m^−^^1^ K^−^^1^), their inherent rigidity creates high compressive modulus (> 100 GPa), resulting in poor interfacial conformity and elevated contact thermal resistance. Conversely, polymer‐dominant formulations (e.g., thermal greases, silicone pads) exhibit excellent compressibility and low contact thermal resistance but suffer from intrinsically low bulk thermal conductivity (< 10 W m^−^^1^ K^−^^1^) due to phonon scattering in amorphous structures [23, 24, 25, 26, 27]. To bridge this gap, liquid metal (LM)‐based solutions have emerged as formidable competitors, leveraging their inherent fluid‐like compliance and high conductivity [28, 29, 30]. However, concerns regarding their high fluidity, potential leakage, and corrosive nature toward metallic components remain critical barriers to long‐term stability. Similarly, vertically aligned metallic architectures, such as copper nanowires, have demonstrated the effectiveness of structural logic in establishing direct heat conduction pathways [31, 32]. Nevertheless, these metallic frameworks often struggle with mechanical resilience in practical assembly.

Consequently, 2D material‐based strategies—particularly those leveraging graphene's exceptional intrinsic properties—are poised as the next frontier for high‐power‐density thermal management [33, 34]. According to recent authoritative roadmaps and reviews [35, 36, 37], maximizing thermal transport efficiency requires vertically oriented graphene architectures. To date, such architectures—fabricated via chemical vapor deposition, magnetic/electric field alignment, or micro‐filament shearing—have been developed to create continuous heat conduction pathways through the composite matrix [38, 39, 40, 41]. However, these traditional assembly methods often suffer from weak interlayer bonding, numerous defects, and high contact thermal resistance. While vertically aligned graphene films exhibit ultrahigh through‐plane thermal conductivity [42], their high compressive modulus (> 60 GPa) translates into prohibitively high stiffness in composite systems. Under normal packaging pressures, the composites struggle to deform sufficiently to conform well to interfaces. This leads to persistently high contact thermal resistance, representing a critical bottleneck limiting their practical cooling efficiency. Therefore, simultaneously achieving ultrahigh through‐plane thermal conductivity and low compressive modulus in TIMs remains a core challenge.

Here, we propose vertically oriented GMPs through laser cutting, vertical assembly, and silicone rubber encapsulation. By optimizing the graphene content to 60 wt.% (GMP60), an unprecedented performance synergy is achieved: a through‐plane thermal conductivity of 565.92 W m^−^^1^ K^−^^1^, a minimal total thermal resistance of 0.028 in^2^ K W^−^^1^, and a compressive modulus of 115.16 kPa, coupled with exceptional stability and fatigue resistance. The GMPs further exhibit outstanding thermal stability (< ± 2.1% thermal conductivity fluctuation after 20 cycles) and mechanical resilience (1000 compression cycles). In practical validation, GMP60 lowers CPU core temperatures by 12°C under full‐load operation and reduces power chip temperatures by 18.2°C.

## 2 Results and Discussion

### 2.1 Fabrication and Characterization of GMPs

**FIGURE 1:**
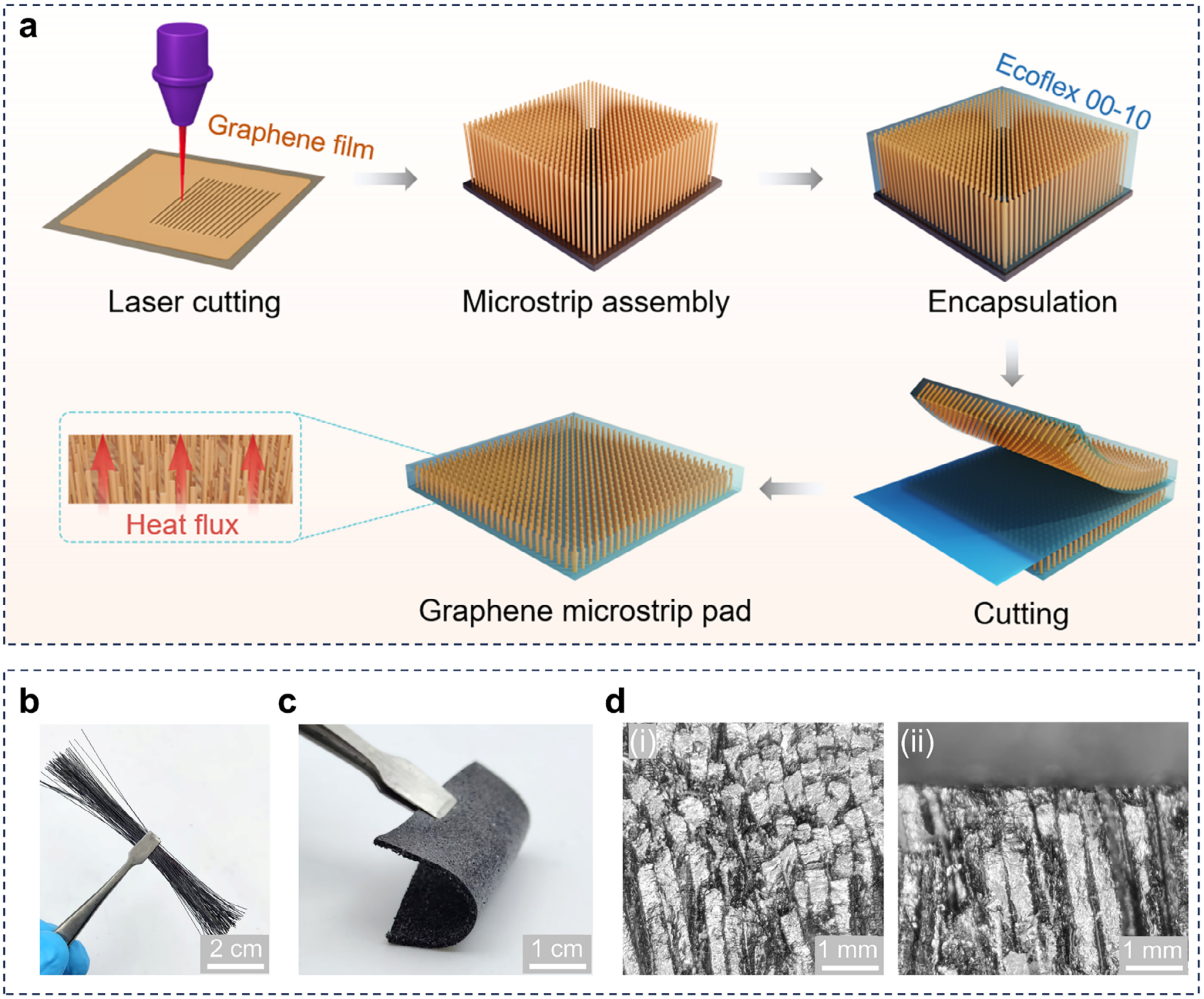
(a) Fabrication process of GMPs. (b) Images of laser‐cut graphene microstrips. (c) Optical photograph of GMP. (d) Surface and cross‐sectional optical microscopy images of GMPs.

The fabrication process of the GMPs involves laser cutting, vertical assembly of graphene microstrips, silicone rubber encapsulation, and slicing, as illustrated in Figure 1a. First, graphene films are laser‐cut into microstrips (Figure 1b). The graphene microstrip retains the same excellent mechanical and thermal properties as the original graphene film (Figure S1). Scanning electron microscopy (SEM) characterization reveals that the graphene microstrips have a width of 200 µm (Figure S2). Secondly, the graphene microstrips are vertically assembled within a square mold and encapsulated using Ecoflex 00–10 silicone rubber. The vertical orientation is stabilized through mechanical self‐confinement and structural interlocking induced by lateral compressive forces. This self‐supporting framework remains intact during the subsequent vacuum‐assisted infiltration, effectively preventing any orientation deviation during the matrix encapsulation and thermal curing stages. Finally, the graphene microstrip/Ecoflex 00–10 composite is sliced into thin pads to obtain the GMPs, shown in Figure 1c. For clarity, samples with 40–80 wt.% graphene microstrip content are labeled GMP40, GMP50, GMP60, GMP70, and GMP80, respectively. The Ecoflex 00–10 silicone rubber imparts excellent flexibility to GMP, allowing it to be easily bent (Figure S3). Furthermore, a strong interfacial adhesion (1.04 MPa) between Ecoflex 00–10 and the graphene microstrips ensures the mechanical stability of GMPs (Figure S4). Optical microscopy images confirm the dense, vertically aligned arrangement of the graphene microstrips within the pad (Figure 1d). This vertically aligned structure facilitates efficient heat conduction in the through‐plane direction. The cutting‐assembly‐infiltration strategy is highly scalable, with process steps that can be transformed into standardized industrial units. It utilizes laser cutting for high precision, mechanical self‐constraint within modular molds to achieve spontaneous vertical alignment, and vacuum‐assisted infiltration for high‐throughput, void‐free matrix impregnation. By leveraging these automated processes, consistent quality in large‐scale manufacturing can be achieved.

**FIGURE 2:**
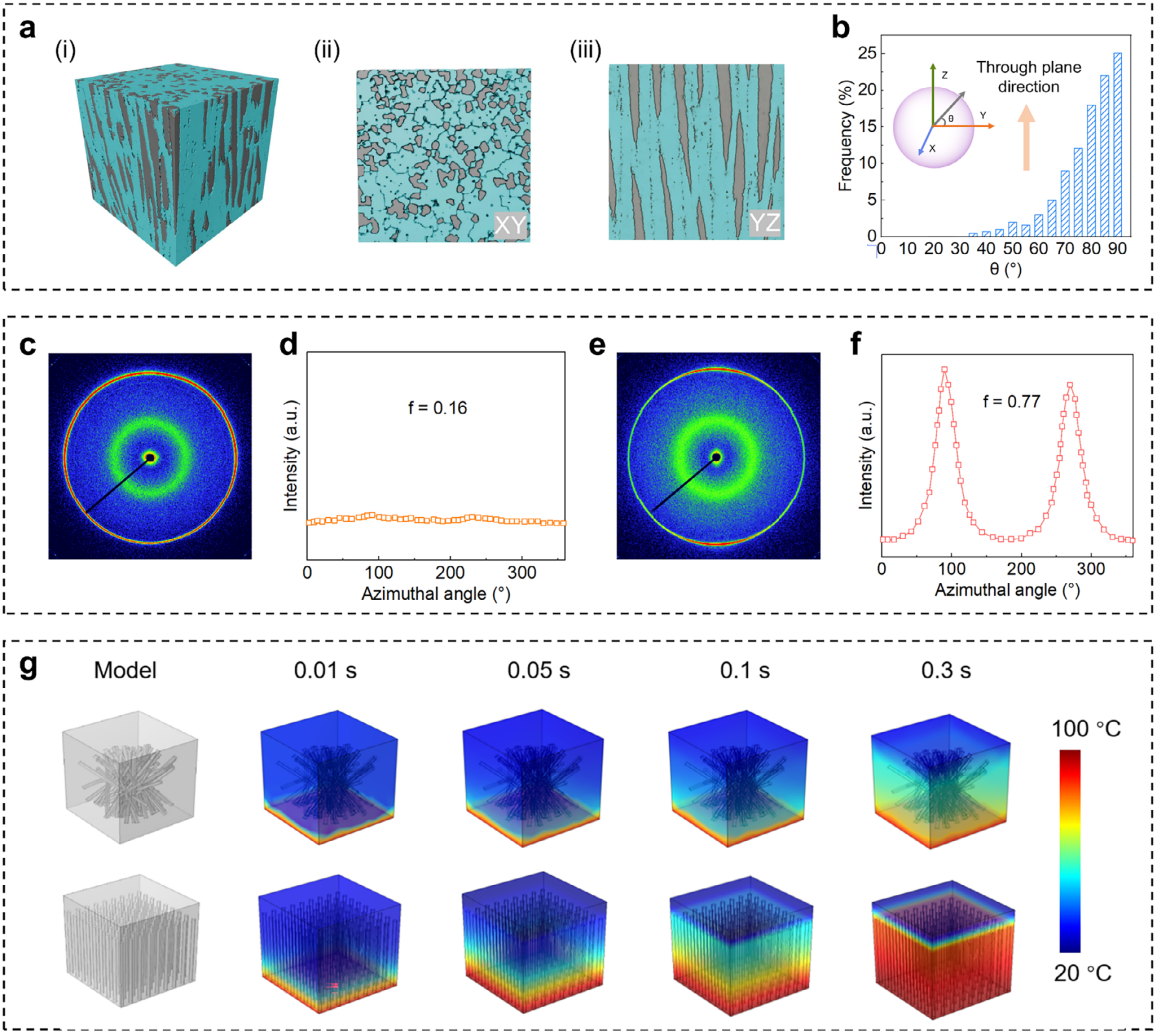
(a) Reconstructed 3D model from micro‐CT characterization of GMP60. (b) Statistical analysis of microstrip angle distribution in GMP60. (c–f) WAXS patterns and azimuthal angle curves of disordered and ordered GMP60. (g) Finite element simulation of heat conduction characteristics in disordered and ordered GMPs.

The orientation architecture of thermally conductive fillers fundamentally governs the heat conduction pathways in TIMs, making precise characterization of microstructural alignment indispensable for understanding their exceptional through‐plane conductivity. Non‐destructive micro‐computed tomography (Micro‐CT) was employed to provide a detailed view of the GMPs. Figure 2a shows a 3D reconstruction of a GMP60, along with cross‐sectional slices in the XY and YZ planes. Quantitative analysis of the tilt angle (θ) distribution was performed for 1000 graphene microstrips within the test volume. The histogram reveals that 86.2% of the microstrips had a θ angle between 70° and 90°, indicating a strong preference for vertical alignment (Figure 2b). This contrasts sharply with the disorderedly assembled GMP60, where over 80% of microstrips had θ angles below 50° (Figure S5). Additionally, the degree of orientation within the GMP60 was assessed using wide‐angle x‐ray scattering (WAXS). Figure 2c displays the scattering pattern from a disorderedly assembled sample. This pattern is nearly isotropic (forming a complete ring), and fitting shows no distinct characteristic peaks (Figure 2d), resulting in a low orientation factor of only 0.16. In contrast, the scattering pattern from the vertically ordered GMP60 exhibits a distinct symmetric arced diffraction pattern (Figure 2e), with significantly higher scattering intensity at the top and bottom positions. Figure 2f shows sharp peaks at 0° and 270°, corresponding to a high orientation factor of 0.77. Collectively, these findings demonstrate that the graphene microstrips within GMP are highly ordered and vertically aligned, providing strong evidence for excellent through‐plane heat conduction capability. The difference in thermal conduction performance between disordered and orderly assembled GMPs was further simulated using the finite element method. Figure 2g illustrates the heat conduction process from a 100°C bottom heat source to the sample top. In the ordered GMP, heat propagates rapidly along vertically aligned graphene microstrips, establishing continuous thermal pathways that minimize interfacial resistance and phonon scattering. This enables complete heat conduction within 0.3 s, achieving uniform temperature distribution across the entire structure. Conversely, the disordered GMP exhibits severely impeded heat conduction. After the same 0.3 s duration, only the bottom region shows a significant temperature increase (> 70°C), while the top sections remain near ambient temperature (< 30°C). Such localized thermal confinement directly manifests the critical role of vertically aligned graphene microstrips in enabling efficient through‐plane conduction.

### 2.2 Thermal and Mechanical Properties of GMPs

**FIGURE 3:**
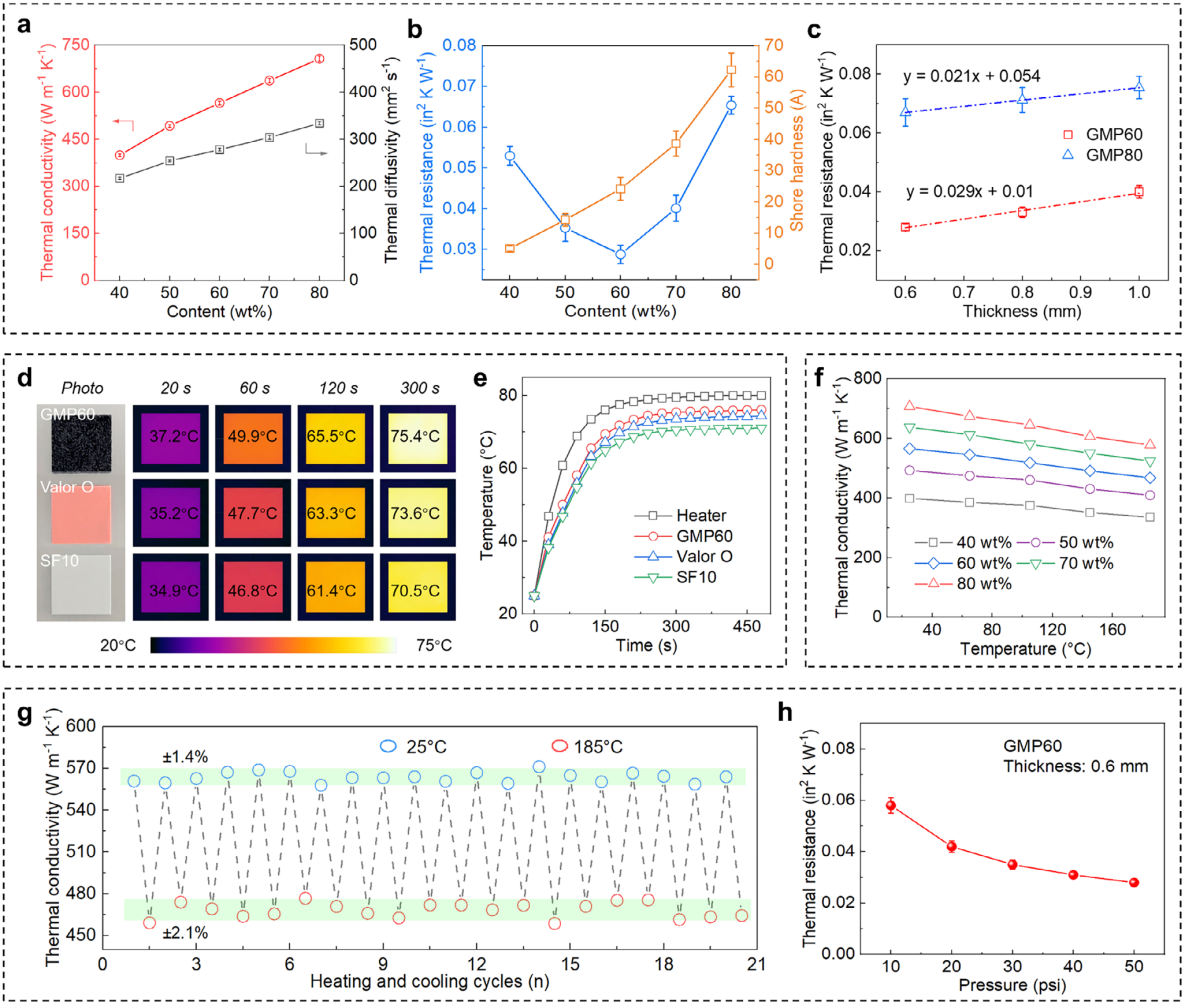
(a) Through‐plane thermal conductivity and thermal diffusivity of GMPs. (b) Total thermal resistance and shore hardness of GMPs. (c) Decoupling bulk thermal resistance and contact thermal resistance using the multi‐thickness method. (d) Thermal infrared images during the heat conduction of different samples. (e) Surface temperature‐time profiles extracted from the IR data. (f) Thermal conductivity of GMPs at different temperatures. (g) Thermal conductivity stability of GMPs during heating‐cooling cycles. (h) Thermal resistance of the GMP60 sample across a pressure range from 10 to 50 psi.

The thermal properties of GMPs were systematically evaluated through through‐plane thermal conductivity and thermal resistance measurements. Thermal diffusivity was determined via laser flash analysis (ASTM E1461), with detailed calculation parameters provided in Table S1. As shown in Figure 3a, through‐plane thermal conductivity increases progressively with graphene microstrip content, reaching 400.46 (40 wt.%), 493.32 (50 wt.%), 565.92 (60 wt.%), 639.3 (70 wt.%), and 707.34 W m^−^^1^ K^−^^1^ (80 wt.%). This ultrahigh thermal conductivity stems primarily from the exceptional in‐plane thermal conductivity of graphene films (1450 W m^−^^1^ K^−^^1^). Interfacial thermal behavior of GMPs reveals a non‐monotonic trend. Total thermal resistance (R_t_) of the 0.6 mm thick samples, measured by guarded hot plate method (ASTM D5470) at 50 psi, decreases from 0.053 to 0.028 in^2^ K W^−^^1^ as graphene microstrip content rises from 40 to 60 wt.%, but surges 2.32‐fold to 0.065 in^2^ K W^−^^1^ at 80 wt.% (Figure 3b).

To clarify the dominant mechanism behind the performance degradation at high filler loadings (80 wt.%), we employed the multi‐thickness method to decouple the bulk thermal resistance (R_b_) and the interfacial contact resistance (R_c_) for both GMP60 and GMP80 at a pressure of 50 psi [43, 44]. As shown in Figure 3c, the R_t_ increases linearly with the increase in sample thickness. Upon increasing the graphene content from 60 to 80 wt.%, the slope of the fitting line decreases from 0.029 to 0.021 (a 27.6% reduction), indicating that the intrinsic bulk thermal conductivity of GMP80 remains superior. Specifically, at a thickness of 0.6 mm, the R_b_ of GMP60 (0.011 in^2^ K W^−^^1^) is lower than that of GMP60 (0.018 in^2^ K W^−^^1^). However, this improvement is completely eclipsed by a 5.4‐fold surge in R_c,_ which jumps from 0.01 to 0.054 in^2^ K W^−^^1^. This decoupling clearly demonstrates that the performance loss at 80 wt.% is exclusively driven by R_c_ rather than R_b_. The significantly higher hardness (63A) and modulus of GMP80 prevent the material from conforming to the microscopic asperities of the contact surfaces under 50 psi, creating a massive thermal barrier at the interface. It is noteworthy that under a standard operating pressure of 50 psi, the k_eff_ of our GMP60 reaches an impressive 33.2 W m^−^^1^ K^−^^1^, which is significantly superior to representative commercial dielectric TIMs (typically < 5 W m^−^^1^ K^−^^1^), such as Chomerics Gap Filler Pad 976, Honeywell TIP 3500, and Henkel TSP 3500. The R_t_ of GMP60 (0.028 in^2^ K W^−^^1^) also outperforms most of the reported TIMs (Table S2).

To further validate the practical superiority of the vertically oriented GMP structure, a real‐time infrared (IR) thermography benchmark was conducted, comparing GMP60 against two state‐of‐the‐art commercial thermal pads: Valor O (Thermalright, 15 W m^−^^1^ K^−^^1^) and Tflex SF10 (Laird, 10 W m^−^^1^ K^−^^1^). All samples were standardized to a thickness of 1 mm to ensure a rigorous comparison of their bulk transport efficiencies. The samples were placed on a uniform heating platform that ramped from 25°C to 80°C. As captured in the IR images (Figure 3d), the top‐surface temperature of the GMP60 sample rose significantly faster than its commercial counterparts. The quantitative heating curves (Figure 3e) reveal that GMP60 reached 50°C within only 60 s, whereas the Valor O and Tflex SF10 pads required 64 and 67 s, respectively. Specifically, at t = 120 s, the surface temperature of GMP60 was recorded at 65.5°C, which is 2.2°C higher than that of the Valor O pad. This real‐world demonstration confirms that the structural logic of GMPs provides a more effective thermal management solution for high‐power‐density electronics than traditional isotropic commercial alternatives.

Beyond rapid thermal conduction, the operational robustness of GMPs under thermal stress is a prerequisite for long‐term practical applications. Thermogravimetric analysis (TGA) initially confirms the inherent thermal stability of the GMP samples, with no significant decomposition observed below 300°C across all samples (Figure S6). This structural integrity further ensures consistent thermal transport performance. Specifically, the thermal conductivity of the GMPs exhibits only a marginal decrease across a wide operating window from 25°C to 185°C (Figure 3f). All GMPs still maintain thermal conductivities above 330 W m^−^^1^ K^−^^1^, demonstrating excellent heat dissipation performance within typical electronic device operating temperatures (< 150°C). Furthermore, the stability of GMPs’ thermal conductivity under thermal shock conditions was also evaluated. After 20 thermal cycles, GMP60 shows only a negligible variation in conductivity at both low and high temperatures (25°C and 185°C). The deviations at 25°C and 185°C are as low as ± 1.4% and ± 2.1%, respectively, highlighting the reliable thermal stability of the GMPs (Figure 3g). The structural uniformity and fabrication consistency were evaluated by testing five independent batches of GMP60. The resulting thermal conductivity values displayed a narrow distribution centered at 563.4 W m^−^^1^ K^−^^1^ with a relative standard deviation (RSD) of 3.88% (Figure S7). This exceptional consistency is attributed to the synergistic effect of automated laser dicing and vacuum‐assisted infiltration, which ensures that the hierarchical graphene pathways are uniformly integrated into the silicone matrix. Such high‐fidelity replication across batches highlights the robustness of our manufacturing logic, suggesting that the GMP architecture can be reliably scaled up without compromising its record‐breaking thermal transport properties 4 In addition, the conformability and performance under low mounting pressures are also vital for delicate electronic components. We have systematically measured the R_t_ of the 0.6 mm thick GMP60 sample across a pressure range from 10 to 50 psi (Figure 3h). Notably, even at a minimum pressure of 10 psi, the GMP60 exhibits a remarkable R_t_ of 0.058 in^2^ K W^−^^1^, providing a robust solution for diverse electronic packaging scenarios.

**FIGURE 4:**
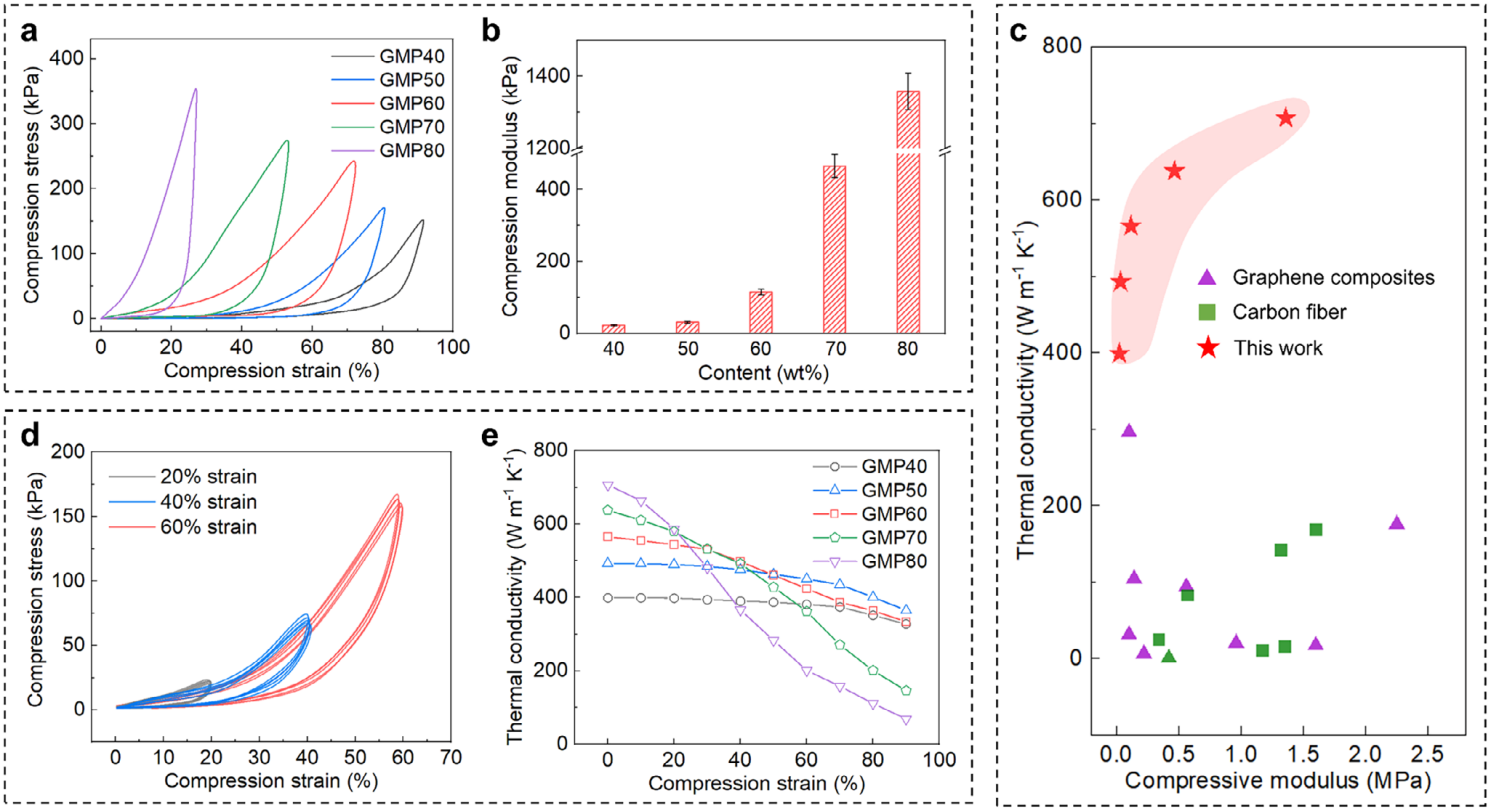
(a) Stress–strain curves of GMPs. (b) Compressive modulus of GMPs. (c) Comparison of mechanical and thermal properties between fabricated GMPs and reported literature data. (d) Cyclic compressive stability of GMP60. (e) Thermal conductivity of GMPs after compression.

In addition to excellent thermal properties, the GMPs also demonstrate outstanding mechanical performance. Due to the significant flexibility of the silicone rubber, the GMPs exhibit commendable compressibility and resilience (Figure S8). Figure 4a presents the stress–strain curves for the compression and release of the GMPs. As the microstrip content increases from 40 to 80 wt.%, the compressive stress of the samples increases, while the achievable strain range decreases (Figure S9). For example, GMP40 shows a compressive stress of 153.21 kPa and a strain range of 0%–92.67%. In contrast, GMP80 exhibited a significantly higher compressive stress of 363.68 kPa but a much smaller strain range of 0%–27.25%. GMP40, GMP50, and GMP60 possess ultralow compressive modulus of 22.63, 31.53, and 115.16 kPa, respectively (Figure 4b). To further elucidate the deformation mechanism of the composite material, we conducted finite element simulations (Figure S10). The simulation results confirmed that the flexible silicone matrix effectively accommodated the displacement of graphene microstrips, providing sufficient volume space for the compressibility of GMPs even at a GMP80. A lower compressive modulus indicates excellent conformability to microscopic surface irregularities, thereby increasing the effective contact area and enhancing thermal conduction efficiency. The GMPs demonstrate unprecedented performance advantages by simultaneously achieving an ultrahigh through‐plane thermal conductivity and an exceptionally low compressive modulus, which outperforms all reported graphene‐based or carbon fiber‐based TIMs (Figure 4c; Table S3). Furthermore, the mechanical stability of the samples was tested. Figure 4d depicts cyclic stress–strain tests on GMP60 under different pressures. During five compression‐release cycles at strains of 20%, 40%, and 60%, the curves for each cycle overlapped significantly, demonstrating remarkable consistency. GMP60 also underwent 1000 cycles of compressive loading at a constant stress of 100 kPa. Results show no significant degradation in the elasticity after cycling (Figure S11), indicating excellent structural fatigue resistance. The structural robustness of the GMPs was evaluated by measuring their thermal conductivity ex situ after being subjected to compressive strains ranging from 0% to 90% (Figure 4e). Following the pre‐compression cycles, the thermal conductivity exhibited a slight decrease with increasing strain, a trend more pronounced in samples with higher graphene content. Importantly, this ex situ characterization confirms that even after an extreme 90% compression, the vertically aligned heat‐conduction pathways remain substantially intact. For instance, GMP40, GMP50, and GMP60 maintain robust thermal conductivities above 320 W m^−^^1^ K^−^^1^ after release, demonstrating their exceptional mechanical‐thermal reliability as high‐efficiency TIMs.

### 2.3 Thermal Performance Validation and Application of GMPs

**FIGURE 5:**
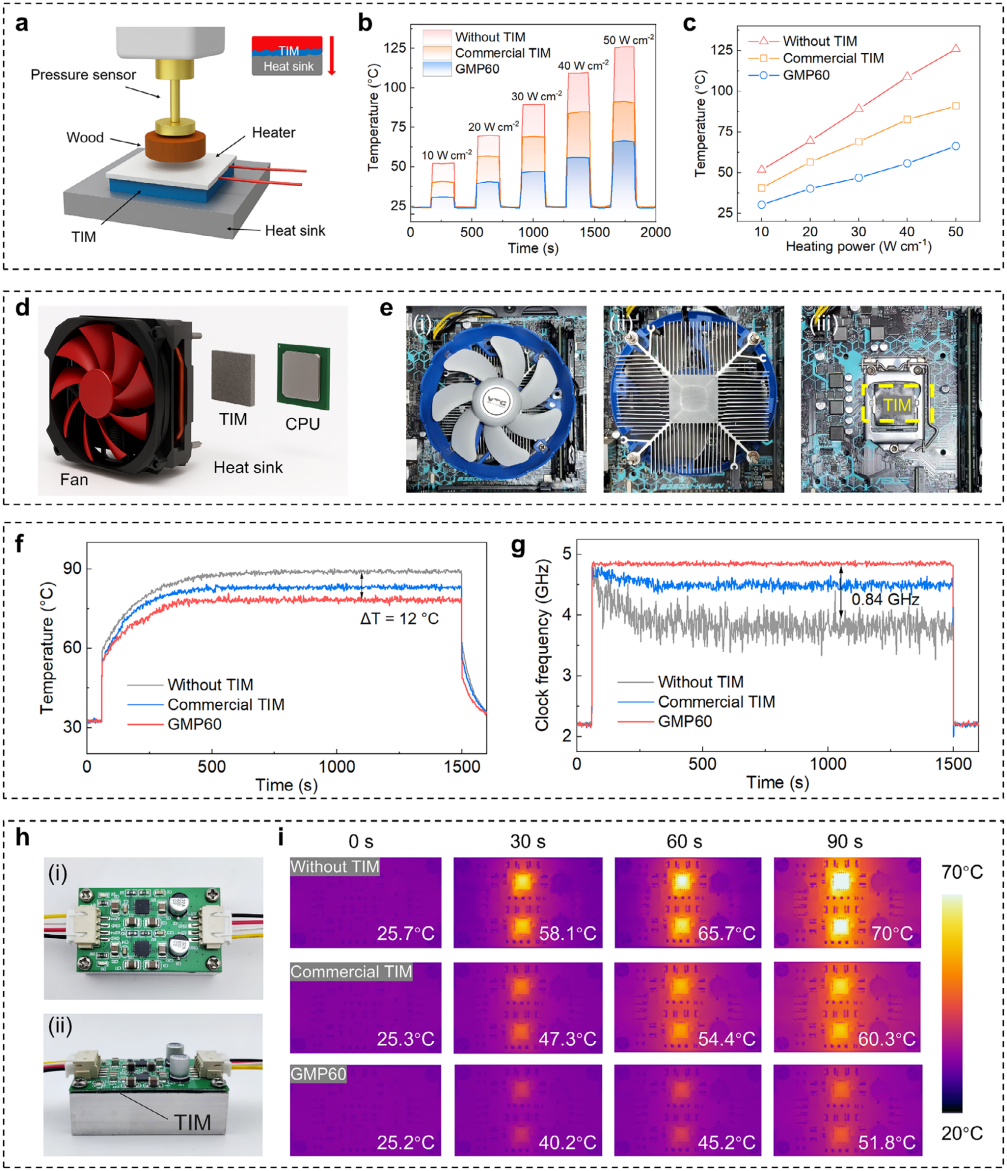
(a) Schematic of thermal conductivity testing for TIMs. (b,c) Temperature comparison of TIMs at varied heater power densities. (d,e) CPU cooling system schematic and physical assembly. (f,g) Temperature and clock frequency evolution in the CPU system with TIMs. (h) Power circuit thermal dissipation using TIMs. (i) IR images of power circuits with TIMs.

To validate the interfacial heat conduction performance of GMPs under practical assembly conditions, a test system was constructed, as shown in Figure 5a. A GMP60 sample measuring 30 × 30 × 1 mm was placed between a ceramic heating element and a heat sink. An assembly pressure of 50 psi was applied using a pressure sensor. Thermal insulating wood blocks were positioned between the pressure sensor and the heater to minimize heat loss and ensure a stable heat source temperature. Heat generated by the heater passed through the TIM to a water‐cooled heat sink. Comparative cooling effects without any TIM, with a commercial TIM (TGP8000PT, k = 8 W m^−^^1^ K^−^^1^), and with GMP60 as the TIM are presented in Figure 5b. As the power density of the ceramic heating element increased from 10 W cm^−^^2^ to 50 W cm^−^^2^, the temperature of the ceramic piece without TIM rose sharply from 52°C to 126°C. Under identical power densities, the temperature of the ceramic heating element using GMP60 as the TIM is lower than when using the commercial TIM (Figure 5c). For instance, at 50 W cm^−^^2^, the temperature with GMP60 was 67.4°C, compared to 89.1°C for the commercial TIM, showing a difference of 21.7°C.

We conducted comprehensive performance tests in real‐world electronic cooling scenarios to validate the practical superiority of GMP60 as TIM. Rising CPU temperatures significantly degrade processor performance, frequently causing thermal throttling, clock speed reductions, and system instability. To assess GMP60's ability in countering these thermal limitations, we established a standardized test configuration (Figure 5d). The TIM was installed between the CPU (Intel Core i5) and an air‐cooled heat sink, as shown in Figure 5e. The CPU undergoes a 100% stress test using AIDA64 software, with real‐time monitoring of core temperature and effective clock frequency. Comparative tests revealed that without TIM, the CPU temperature peaks at 89.3°C, which is reduced to 83.7°C with commercial TIM and further drops to 77.3°C using GMP60 (Figure 5f). The CPU thermal throttling mechanism was clearly demonstrated in our tests. Higher temperatures trigger clock frequency reduction, while GMP60's superior cooling enabled 0.84 GHz higher sustained clock speeds (Figure 5g). In a separate demonstration using a linear power supply circuit (Figure 5h), IR imaging showed the power chip reaching 70°C within 90 s without TIM (Figure 5i). Temperatures are significantly lower using either the commercial TIM or GMP60 at various time points (Figure S12). After 90 s of operation, the maximum chip temperature was 60.3°C with the commercial TIM, but only 51.8°C with GMP60. This represents reductions of 18.2°C and 8.5°C compared to no TIM and commercial TIM, respectively. These results conclusively demonstrate GMP60's exceptional thermal management capabilities that outperform conventional TIMs in real electronic cooling applications.

## 3 Conclusion

In summary, we developed vertically aligned GMPs via laser cutting, vertical microstrip assembly, and silicone rubber encapsulation. This strategy enables precise tuning of thermomechanical properties by varying graphene microstrip content (40–80 wt.%). Ultrahigh through‐plane thermal conductivity (400.46–707.34 W m^−^^1^ K^−^^1^) and ultralow compressive modulus (22.63–115.16 kPa) are simultaneously achieved, overcoming the traditional trade‐off between thermal and mechanical performance in TIMs. By optimizing the graphene content to 60 wt.% (GMP60), we minimized total thermal resistance to 0.028 in^2^ K W^−^^1^ (50 psi). In addition, the GMPs also exhibited outstanding thermal stability and mechanical resilience. The thermal conductivity varies by less than ± 2.1% over 20 thermal cycles (25°C–185°C) and remains above 330 W m^−^^1^ K^−^^1^ at typical electronics operating temperatures (< 150°C). Mechanical resilience is demonstrated through 1000 compression cycles without significant degradation, ensuring long‐term reliability in dynamic interfaces. In practical validation, GMP60 lowered CPU core temperatures by 12°C under full‐load operation and reduced power chip temperatures by 18.2°C versus commercial TIMs. This work provides a strategy to overcome the thermal management bottleneck in high‐power‐density electronics, demonstrating significant potential for next‐generation devices.

## 4 Experimental Section

### 4.1 Fabrication of the GMPs

First, an 80‐µm‐thick graphene film (purchased from Fuxitech) was laser‐cut into strip‐like microstrips with a width of 200 µm and a length of 1 cm. Next, 9 g of these microstrips were vertically packed into a square PET mold (3 × 3 × 1 cm). Subsequently, Part A and Part B of the Ecoflex 00–10 solution (mixed at a 1A:1B weight ratio) were stirred thoroughly until homogeneous, and 6 g of the mixture was poured into the mold. By adjusting the mass ratio of microstrips to elastomer solution, GMPs with varying microstrip content could be obtained. The mold was then placed under vacuum for 30 min to remove air bubbles. After degassing, the mold was transferred to a drying oven and cured at 80°C for 2 h. Finally, by employing the ultrasonic vibrating blade slicing technique, a minimum thickness of 0.6 mm was successfully achieved. Consequently, the cured graphene microstrip/Ecoflex blocks were precisely sliced to yield the thermal pads.

### 4.2 Characterization

Optical microscope images of the samples were acquired using an optical microscope (Olympus BX53). SEM images of the sample surface and cross‐section were obtained using a field emission scanning electron microscope (Zeiss Gemini 300). The 3D structure of the samples was characterized using a micro‐CT scanner (Skyscan 1275). TIMs’ orientation was quantified by WAXS (Xuess 2.0). The thermal diffusion coefficients and specific heat capacities of the samples were determined using a laser flash apparatus (LFA 467) and a differential scanning calorimeter (DSC 214), respectively. The total thermal resistance of the samples was measured using a steady‐state heat flow meter thermal conductivity tester (LW‐9389). IR images were captured using a thermal imaging camera (FOTRIC 246 m). The mechanical properties of the samples were tested using a universal testing machine (UH6502).

## Conflicts of Interest

The authors declare no conflicts of interest.

## Supporting information




**Supporting File**: advs75359‐sup‐0001‐SuppMat.docx.

## Data Availability

The data that support the findings of this study are available from the corresponding author upon reasonable request.
