# Supplementary material for: Highly Vertically Oriented Graphene Microstrip Pads With Ultrahigh Through‐Plane Thermal Conductivity and Ultralow Compressive Modulus for Efficient Heat Dissipation
Source: Adv Sci (Weinh). 2026 Apr 17;13(39):e75359. doi: 10.1002/advs.75359 (PMC13335742; doi:10.1002/advs.75359)
Supplement: Supplementary file 1 — Supporting File: advs75359‐sup‐0001‐SuppMat.docx. [file ADVS-13-e75359-s001.docx]

Supporting Information

Highly Vertically Oriented Graphene Microstrip Pads with Ultrahigh Through-plane Thermal Conductivity and Ultralow Compressive Modulus for Efficient Heat Dissipation

Xu Ran†, Yaru Wang†, Sijia Wu, Hejun Wang, Junhao Shen, Litao Sun, Xing Wu*, and Hengchang Bi*

† These authors contributed equally to this work.

X. Ran, Y. R. Wang, S. J. Wu, H. J. Wang, J. H. Shen, Prof. X. Wu, Prof. H. C. Bi

In Situ Devices Center, School of Integrated Circuits, East China Normal University, 200241, Shanghai, P. R. China
Prof. L. T. Sun

SEU-FEI Nano-Pico Center, Key Laboratory of MEMS of Ministry of Education, Collaborative Innovation Center for Micro/Nano Fabrication, Device and System, Southeast University, Nanjing 210096, P. R. China

E-mail: xwu@cee.ecnu.edu.cn; [hcbi@cee.ecnu.edu.cn](mailto:hcbi@cee.ecnu.edu.cn)

**Supporting Methods**

**Characterization of thermal properties**

To measure the thermal conductivity of the GMP samples, a laser flash method was employed, with the GMPs being subjected to thorough thermal characterization. During this process, a brief but intense pulse of light from a laser acted as the heat source, rapidly heating the exposed surface of the sample. Simultaneously, a high-precision infrared detector monitored and recorded the temperature changes on the opposite, non-exposed side of the sample. This approach allowed for a detailed understanding of the heat conduction dynamics within the GMP materials. The thermal diffusivity $\alpha$ of the GMP samples is calculated using the following equation:

$\alpha=\frac{1.38d^{2}}{\pi^{2}t_{1/2}}$ (1)

where $d$ is the thickness of the sample, and $t_{1/2}$ is the time that takes for the sample to heat to one half of the maximum temperature on the rear surface. This parameter provides insight into how rapidly heat spreads through the material. Subsequently, the thermal conductivity $k$ is then calculated by:

$k=\alpha\rho c_{p}$ (2)

Here, $\rho$ denotes the density of the material, and $c_{p}$ is the specific heat capacity. The thermal conductivity is a crucial metric, indicating the material's ability to conduct heat efficiently. By leveraging this method, the thermal conductivity of the GMP samples was accurately determined, revealing their exceptional heat conduction capabilities. This comprehensive thermal characterization ensures a deep understanding of the thermal performance of the GMPs, facilitating their application in advanced thermal management systems.

**Calculation of Orientation Factor for GMP Samples**

To evaluate the degree of orientation within the GMP samples, Wide-Angle X-ray Scattering (WAXS) measurements were conducted using a Xenocs WAXS System. An incident Cu-Kα X-ray beam with a wavelength of 0.154 nm was employed, directed parallel to the sheet plane of the GMP samples. The distance between the samples and the detector was precisely set at 1185 mm to ensure accurate data collection.

The GMP samples were prepared as thin slices, measuring 50 mm in length, 50 mm in width, and 1 mm in thickness. These dimensions facilitated comprehensive scattering analysis while maintaining the structural integrity of the samples. The WAXS measurements were carried out on multiple samples to ensure reproducibility and statistical significance. The scattering patterns were meticulously collected using a PILATUS 300k detector, capable of high-resolution data acquisition.

Foxtrot software was utilized to analyze the 2D WAXS data, providing azimuthal angle *φ* plots that revealed the orientation distribution of the graphene microstrips within the GMP samples. The degree of orientation, also known as the orientation factor *f*, was calculated using the following equation:

$f=\frac{180-FWHM}{180}\times100\%$  (3)

Here, FWHM represents the full width at half maximum of the peak in the azimuthal intensity profile derived from the φ plots. This metric quantifies the degree to which the graphene microstrips are aligned within the GMP samples, with higher values indicating better orientation. Through this meticulous WAXS analysis, the orientation factor of the GMP samples was determined, providing valuable insights into their microstructural alignment and, consequently, their thermal transport properties.

**Characterization of Thermal Resistance**

To evaluate the total thermal resistance (*R_t_*_​_) of the GMP samples, a steady-state heat flux method (ASTM D5470 standard) was employed under controlled conditions of 50 psi pressure. During the test, the sample was sandwiched between two precision-machined copper rods, with the upper rod heated to 80 °C (*T_h_*) and the lower rod cooled to 55 °C (*T_c_*) via circulating water. The *R_t_*_​_ is calculated using the following equation:

$R_{t}=S_{TIM} \times\frac{2(T_{h}-T_{c})}{Q_{h}+Q_{c}}$ (4)

where $S_{TIM}$ is the heat conduction area of the TIM sample. $Q_{h}$ and $Q_{c}$ are the heat fluxes of the heating unit and cooling unit.

The $R_{t}$ reflects the real performance of a TIM in applications since it takes into account both the thermal conductivity ($K_{TIM}$) and thermal contact resistance ($R_{c}$) at the interfaces. The $R_{t}$ values of samples with different graphene microstrip loadings were measured by the steady-state heat flux method. *R_c_* was calculated by measuring $R_{t}$ of samples according to the equation:

$R_{c}={R_{t}-R}_{TIM} = R_{t}- \frac{H}{K_{TIM}}$ (5)

where $R_{TIM}$is the bulk thermal resistance and $H$ is the thickness of samples. Note that the $K_{TIM}$ values are similar to the thermal conductivities tested by the laser flash method.

**Finite Element Simulation of Thermal Transport**

A finite element model using COMSOL Multiphysics was constructed to investigate the critical role of microstructural alignment in the heat conduction performance of graphene microstrip pads (GMPs), comparing vertically aligned structures with disorderedly assembled controls. The simulation framework rigorously replicated experimental conditions using a 1 cm³ (10 mm × 10 mm × 10 mm) cube sample geometry. The filler architecture comprised graphene microstrips with dimensions of 200 μm in width and 80 μm in thickness. For the ordered GMP case, the microstrips were vertically aligned with a tilt angle θ = 90°. In contrast, the disordered control featured microstrips randomly oriented with θ uniformly distributed between 0° and 90°. Material properties included graphene microstrips with a thermal conductivity of 1450 W m⁻¹ K⁻¹ and Ecoflex 00-10 with a conductivity of 0.2 W m⁻¹ K⁻¹, at volume fractions of 40% and 60%, respectively. The boundary conditions applied a constant 100°C temperature to the bottom surface acting as the heat source, while the top surface was set to convective cooling with an ambient temperature of 25 °C, and the sides were thermally insulated.

A free tetrahedral mesh with boundary layer refinement at interfaces was employed, featuring an adaptive element size down to 50 μm for the microstrips. The time-dependent study (0–0.3 s) utilized an implicit backward differentiation formula (BDF) solver. Key results visualized in Figure 2g showed distinct temperature evolution. At 0.3 s, heat propagated rapidly along the vertical microstrips in the ordered GMP, leading to a uniform temperature distribution with the top surface reaching 85 °C, demonstrating efficient through-plane conduction. Conversely, the disordered control exhibited severe heat confinement near the bottom surface (T > 90 °C) while the top remained near 30°C, indicating obstructed thermal pathways and significant phonon scattering at interfaces due to microstrip misalignment.

**Supporting Figures**


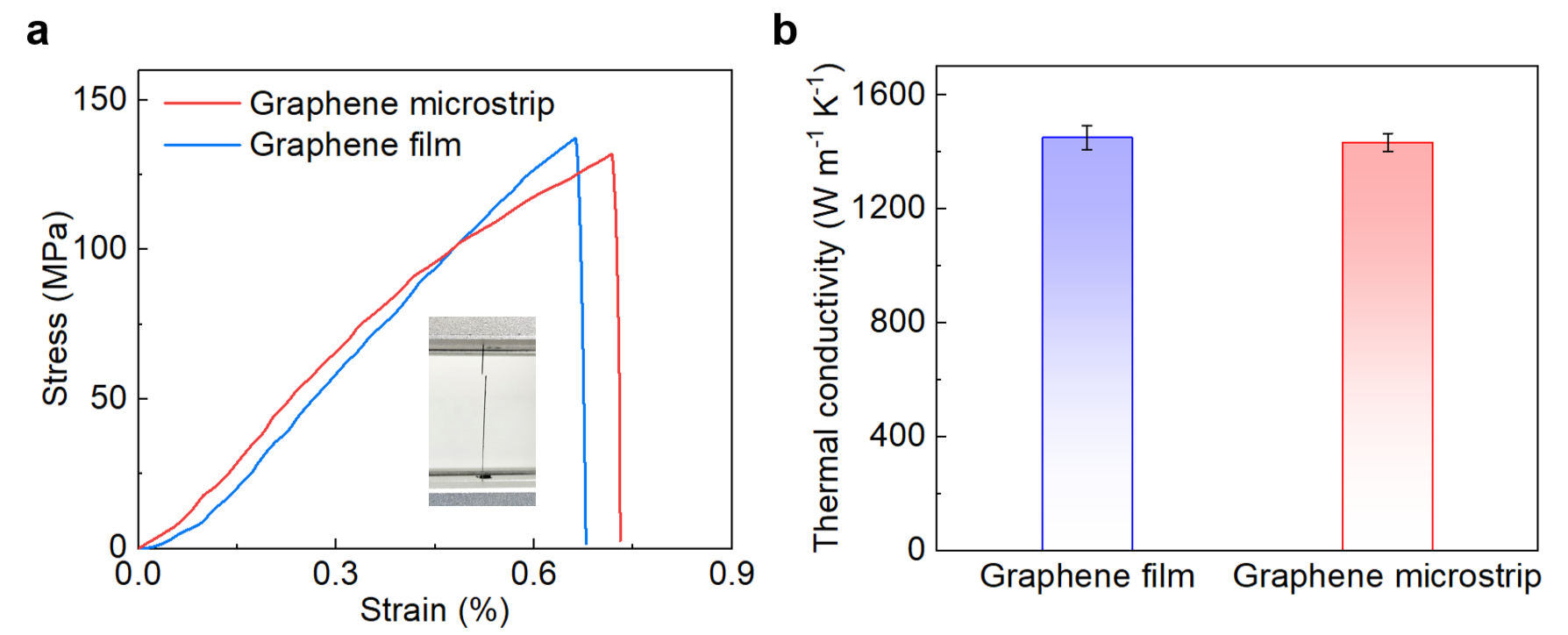


**Figure S1.** a) Stress-strain curves of graphene film and microstrip. b) Thermal conductivity of graphene film and microstrip. Results demonstrate that laser-cut graphene microstrips retain excellent mechanical and thermal properties.


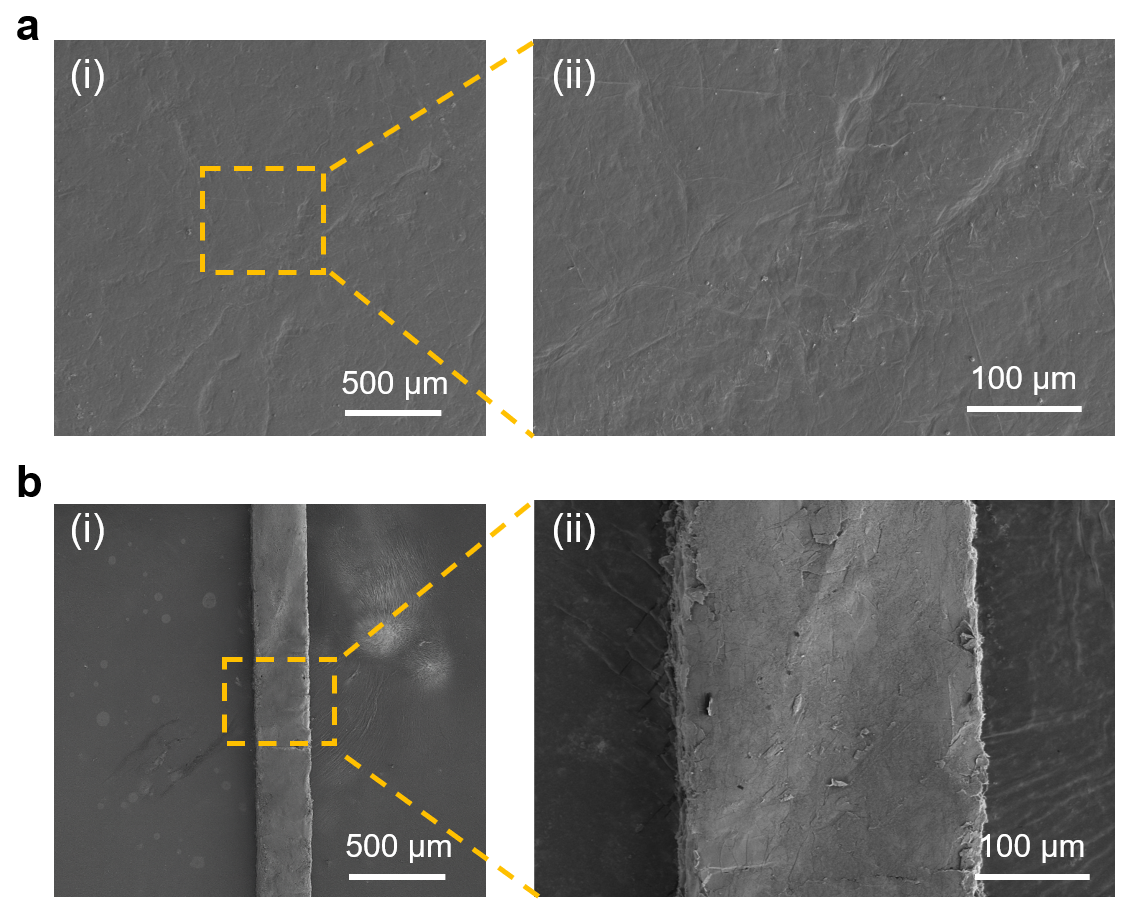


**Figure S2.** a) Surface SEM images of a graphene film. b) SEM images of a graphene microstrip. The laser-cut graphene microstrip exhibits a microstrip-like microstructure, with a width of 200 μm and a thickness of 80 μm.


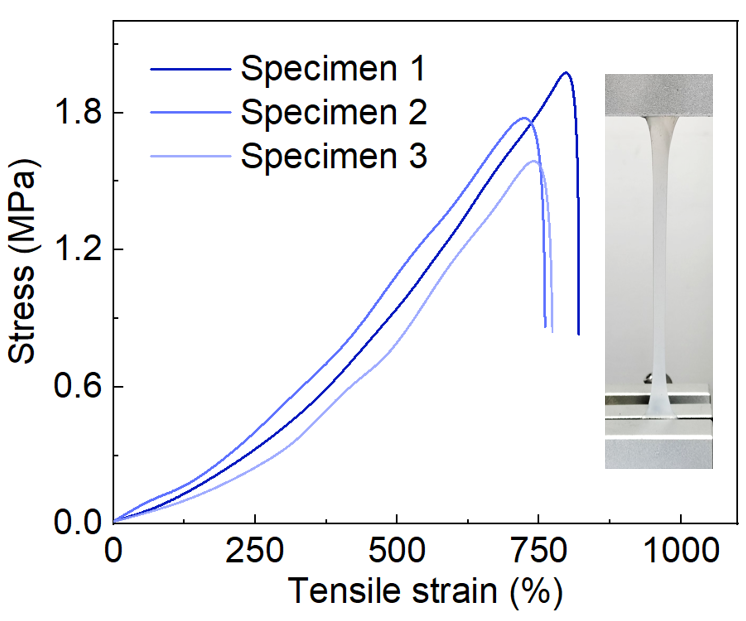


**Figure S3.** Stress-strain curves of three typical Ecoflex 00-10 films. The material exhibits an exceptionally wide strain range (up to 784%) and a fracture stress of 1.8 MPa. These properties contribute to the extremely low compressive modulus of GMPs.


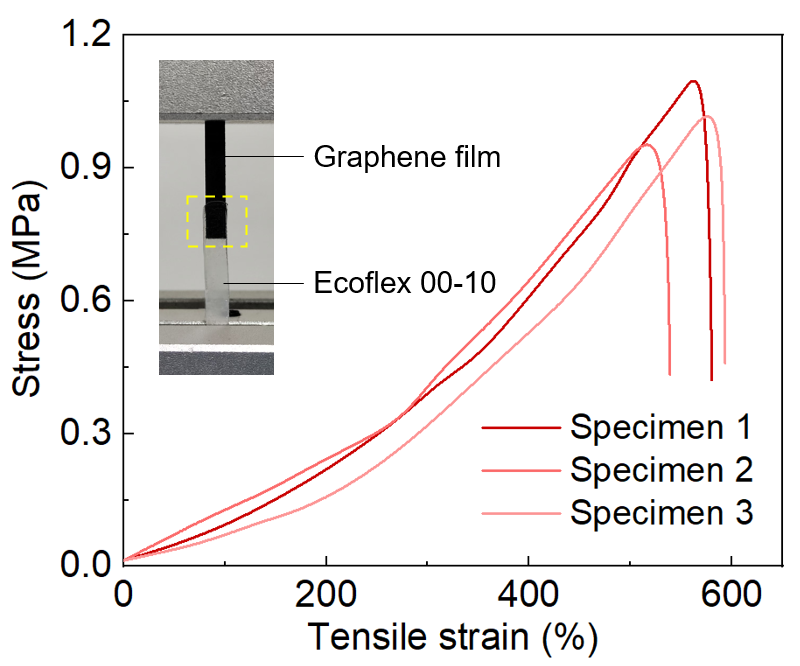


**Figure S4.** Interfacial bonding strength between graphene film and Ecoflex 00-10 elastomer. The graphene film (10 × 5 mm) is encapsulated and cured within the elastomer. The interfacial fracture strength reached 1.04 MPa under longitudinal tension, ensuring mechanical stability in GMPs.


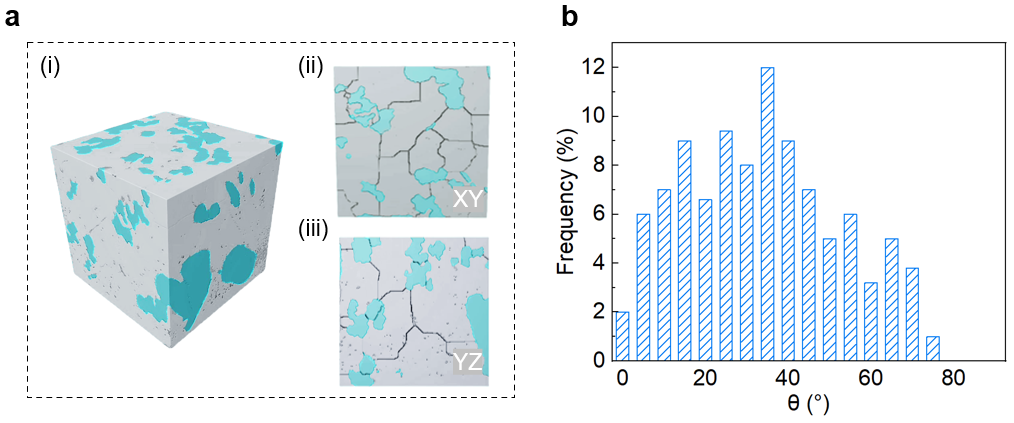


**Figure S5.** a) 3D rendering of a disorderedly assembled GMP with cross-sectional views in the XY and YZ planes. b) Distribution of orientation angles (θ) in disorderedly assembled GMPs, with >80% of graphene microstrips exhibiting θ < 50°.


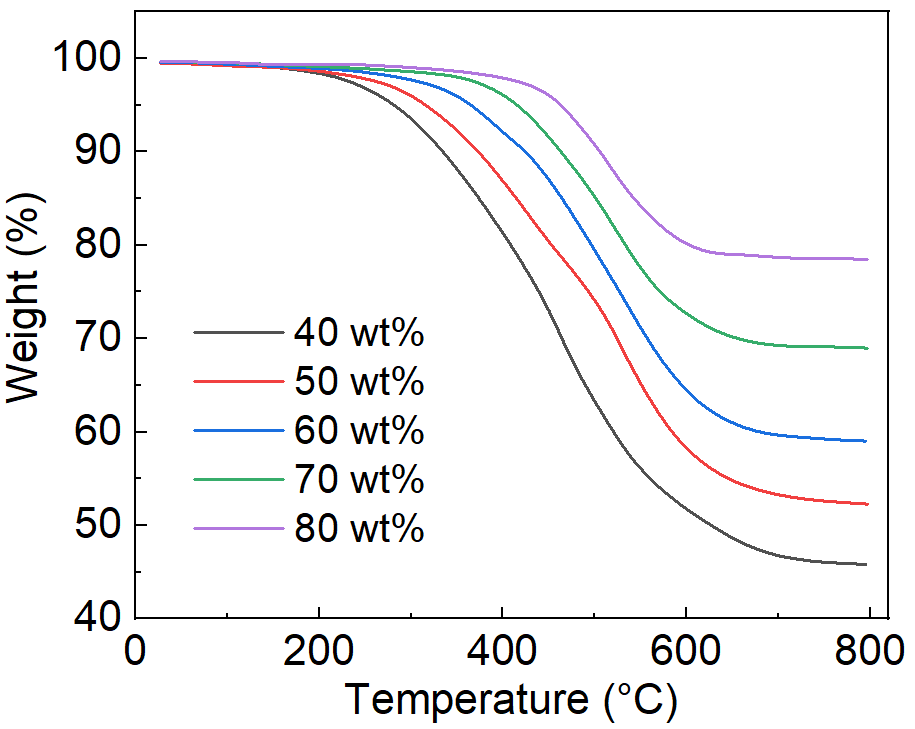


**Figure S6.** Thermal stability of samples in the range of 20–800 °C. Graphene microstrip substantially enhances thermal decomposition resistance of GMPs. The onset temperature for major weight loss increased from 300 °C (GMP40) to 450 °C (GMP80).


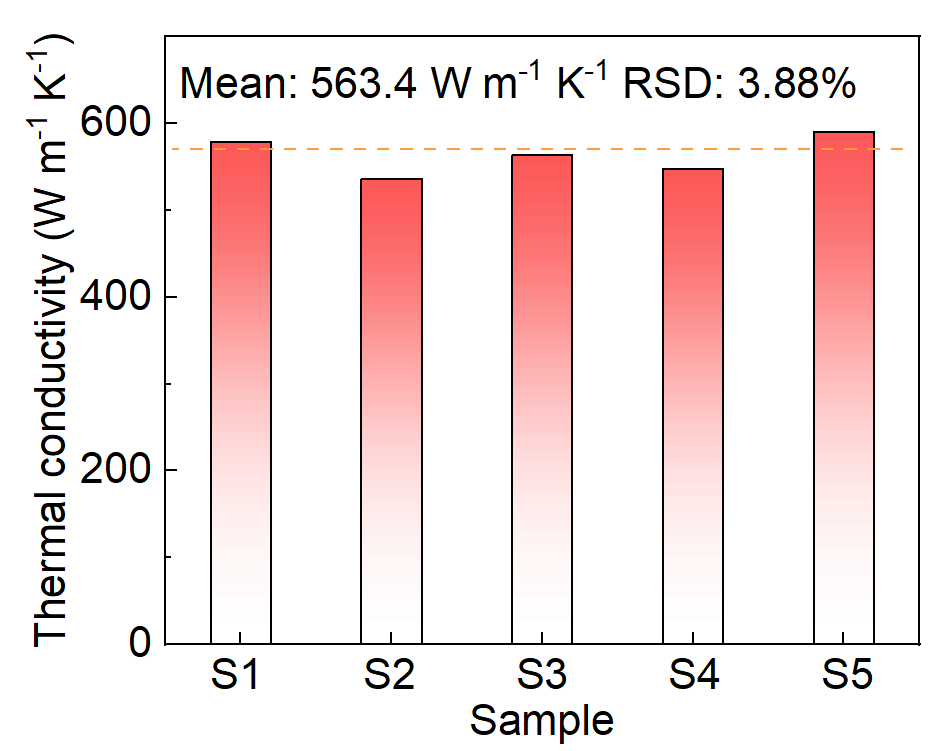


**Figure S7.** Statistical analysis of the fabrication consistency for GMP60 samples. GMP60 demonstrated excellent fabrication consistency across five independent batches, with an arithmetic mean (Mean) thermal conductivity of 563.40 W m⁻¹ K⁻¹ and a RSD of only 3.88% (Standard Deviation, SD = 21.88 W m⁻¹ K⁻¹).


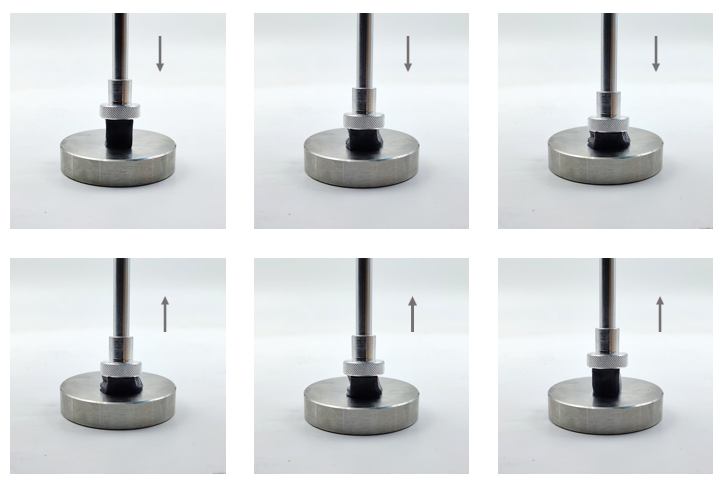


**Figure S8.** Compression-recovery behavior of a GMP60 (10 × 10 × 10 mm) under 50% compressive strain. The material demonstrates complete recovery to its original height after stress release without permanent deformation.


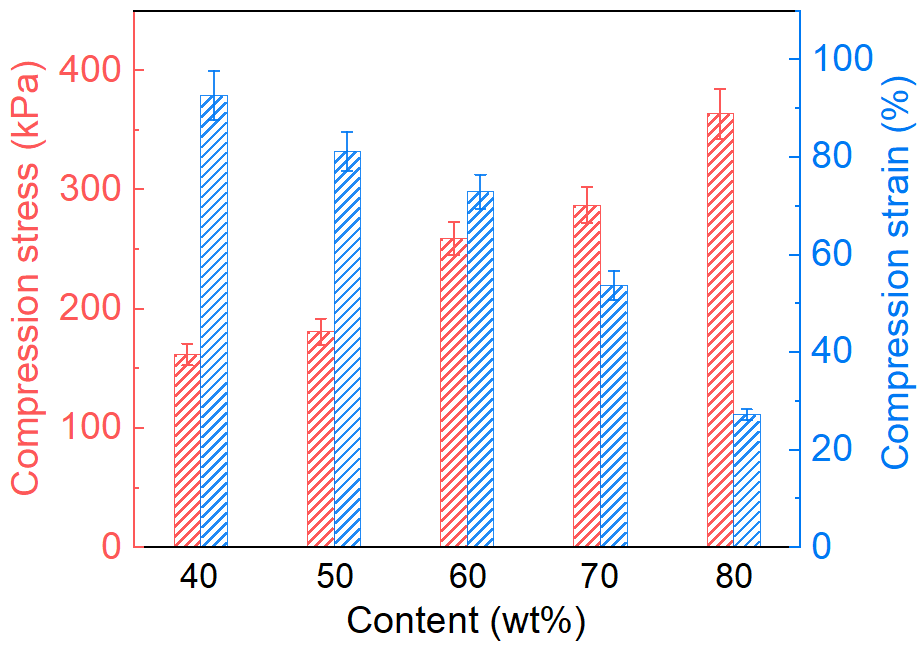


**Figure S9.** Compressive stress-strain behavior of GMPs with 40–80 wt% graphene microstrip content. All compositions demonstrate complete height recovery after stress release. The maximum recoverable compressive strains are 92.67%, 83.70%, 73.21%, 54.34%, and 27.25% for samples containing 40, 50, 60, 70, and 80 wt% graphene microstrips, respectively.


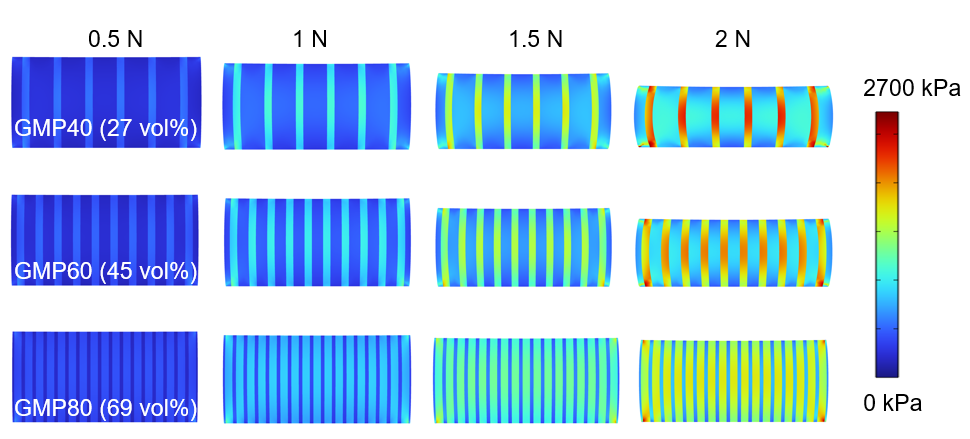


**Figure S10.** Finite element method (FEM) simulation of the mechanical response of GMP composites under vertical compression. To visualize this mechanism, we performed FEM simulations using COMSOL Multiphysics 6.3. A representative volume element of the GMP composite (2 × 2 × 1 mm) featuring vertically aligned graphene microstrips (1 × 0.2 × 0.08 mm). Cross-sectional views showing the stress distribution and corresponding deformation at varying compressive loads (0.5, 1, 1.5, 2 N). The simulation reveals that the synergistic deformation of the low-modulus silicone matrix and the high-aspect-ratio graphene strips allows for substantial vertical displacement. Under vertical compression, the composite does not behave as a monolithic rigid block. Instead, the compliant silicone matrix acts as a flexible buffer, allowing the graphene microstrips to undergo coordinated micro-buckling or tilting. Even at high volume fractions, the silicone matrix provides enough volumetric redundancy to prevent structural locking, ensuring the high compressibility of the TIM.


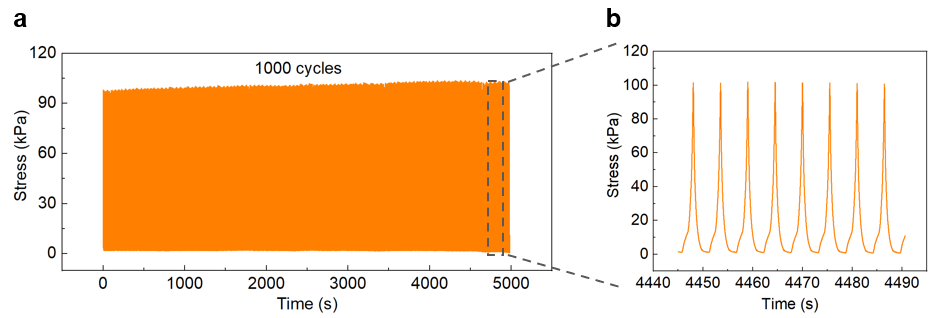


**Figure S11.** Cyclic compression testing of GMP60 under 100 kPa stress at 50% strain for 1000 loading-unloading cycles. Results indicate stable mechanical properties without significant variation after 1000 cycles, demonstrating robust fatigue resistance.


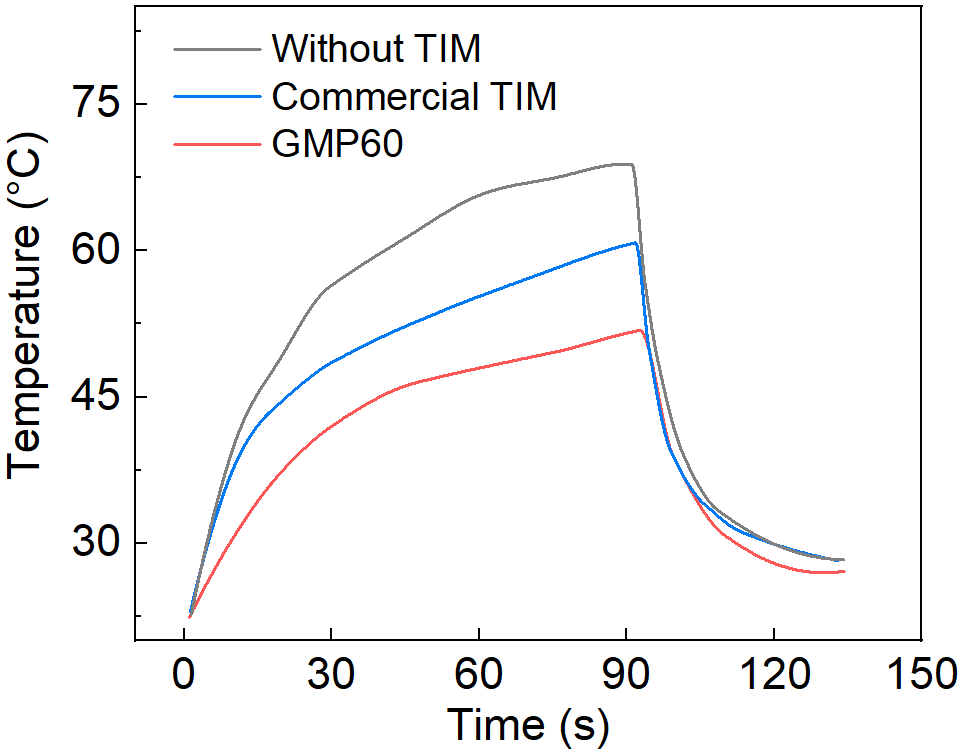


**Figure S12.** Comparison of power chip temperatures under three thermal management conditions: without TIM, with commercial TIM, and with GMP60 during 90 s of operation. At 90 s, the peak temperature reached 60.3 °C with commercial TIM, while GMP60 achieved 51.8 °C, representing reductions of 18.2 °C and 8.5 °C compared to no TIM and commercial TIM, respectively.

**Table S1.** Parameters used for thermal conductivity calculations in GMPs with varying graphene microstrip contents.

| **Microstrip contents (wt%)** | **Heat capacity (kJ kg^-1^ K^-1^)** | **Density (g cm^-3^)** | **Thermal diffusivity (mm^2^ s^-1^)** | **Thermal conductivity (W m^-1^ K^-1^)** |
| --- | --- | --- | --- | --- |
| 40 | 1.26 | 1.46 | 217.69 | 400.46 |
| 50 | 1.22 | 1.59 | 254.32 | 493.32 |
| 60 | 1.19 | 1.71 | 278.11 | 565.92 |
| 70 | 1.14 | 1.84 | 304.78 | 639.3 |
| 80 | 1.08 | 1.96 | 334.16 | 707.34 |

**Table S2.** The total thermal resistance of GMPs compared with existing TIMs in the literature.

| **Materials** | **Thickness (mm)** | **Stress (psi)** | **Total thermal resistance (in^2^ K W^-1^)** | **Reference** |
| --- | --- | --- | --- | --- |
| GNW-TIM | 0.12 | 60 | 3.67 | (1) |
| AGLF-TIMs | 1 | 35 | 7.08 | (2) |
| GHGP | 0.2 | 30 | 2.76 | (3) |
| VGA-TIM | 1.2 | 60 | 2.82 | (4) |
| GF-TIM | 1 | 15 | 14.26 | (5) |
| VSCG/PDMS | 0.3 | 50 | 4.09 | (6) |
| LM-VAGM | 0.8 | 30 | 0.63 | (7) |
| GFR-TIM | 2 | 10 | 0.77 | (8) |
| CVGNPs/PVA | 0.3 | 100 | 7.59 | (9) |
| Al_2_O_3_/BN | 3 | 5.8 | 2.19 | (10) |
| GMP40 | 0.6 | 50 | 0.053 | This work |
| GMP50 | 0.6 | 50 | 0.035 | This work |
| GMP60 | 0.6 | 50 | 0.028 | This work |
| GMP70 | 0.6 | 50 | 0.04 | This work |
| GMP80 | 0.6 | 50 | 0.065 | This work |

**Table S3.** Comparative analysis of thermal and mechanical performance between GMPs and state-of-the-art TIMs reported in the literature.

| **Materials** | **Thermal conductivity (W m^-1^ K^-1^)** | **Compressive modulus (MPa)** | **Reference** |
| --- | --- | --- | --- |
| CVD graphene/PDMS | 0.96 | 20.4 | (1) |
| Graphene microstrip/PDMS | 0.57 | 82.4 | (5) |
| Carbon nanotubes | 0.34 | 24.37 | (6) |
| Graphene composites | 2.25 | 176 | (7) |
| Graphene foam roll | 1.6 | 17.42 | (8) |
| Graphene and PI/PDMS | 0.56 | 94 | (11) |
| Hyperbolic graphene/PDMS | 0.1 | 31.6 | (12) |
| Graphene flake/silicone gel | 0.1 | 296.24 | (13) |
| Carbon microstrip/RTV1010 | 1.35 | 15.3 | (14) |
| Carbon microstrip/PDMS | 1.17 | 10.5 | (15) |
| Graphene/PDMS | 0.42 | 0.83 | (16) |
| GS/EP | 0.14 | 104.6 | (17) |
| Epoxy/TAGA | 0.22 | 6.57 | (18) |
| Carbon microstrip/PDMS | 1.32 | 141.57 | (19) |
| Carbon microstrip | 1.6 | 168.4 | (20) |
| GMP40 | 400.46 | 0.02 | This work |
| GMP50 | 493.32 | 0.03 | This work |
| GMP60 | 565.92 | 0.12 | This work |
| GMP70 | 639.3 | 0.46 | This work |
| GMP80 | 707.34 | 1.36 | This work |

References

[1] Q. Yan, F. E. Alam, J. Gao, W. Dai, X. Tan, L. Lv, J. Wang, H. Zhang, D. Chen, K. Nishimura, L. Wang, J. Yu, J. Lu, R. Sun, R. Xiang, S. Maruyama, H. Zhang, S. Wu, N. Jiang, C. Lin, *Adv. Funct. Mater.* **2021**, *31*, 2104062.

[2] K. Huang, S. Pei, Q. Wei, Q. Zhang, J. Guo, C. Ma, H.-M. Cheng, W. Ren, *ACS Nano* **2024**, *18*, 23468.

[3] J. Gao, Q. Yan, L. Lv, X. Tan, J. Ying, K. Yang, J. Yu, S. Du, Q. Wei, R. Xiang, Y. Yao, X. Zeng, R. Sun, C.-P. Wong, N. Jiang, C.-T. Lin, W. Dai, *Chem. Eng. J.* **2021**, *419*, 129609.

[4] S. Xu, T. Cheng, Q. Yan, C. Shen, Y. Yu, C. Lin, F. Ding, J. Zhang, *Adv. Sci.* **2022**, *9*, 2200737.

[5] J. Lu, X. Ming, M. Cao, Y. Liu, B. Wang, H. Shi, Y. Hao, P. Zhang, K. Li, L. Wang, P. Li, W. Gao, S. Cai, B. Sun, Z.-Z. Yu, Z. Xu, C. Gao, *ACS Nano* **2024**, *18*, 18560.

[6] H. Yu, L. Peng, C. Chen, M. Qin, W. Feng, *Nano-Micro Lett.* **2024**, *16*, 198.

[7] W. Dai, X.-J. Ren, Q. Yan, S. Wang, M. Yang, L. Lv, J. Ying, L. Chen, P. Tao, L. Sun, C. Xue, J. Yu, C. Song, K. Nishimura, N. Jiang, C.-T. Lin, *Nano-Micro Lett.* **2022**, *15*, 9.

[8] Y. Chen, K. Pang, X. Liu, K. Li, J. Lu, S. Cai, Y. Liu, Z. Xu, C. Gao, *Carbon* **2023**, *212*, 118142.

[9] S. Cheng, X. Guo, P. Tan, M. Lin, J. Cai, Y. Zhou, D. Zhao, W. Cai, Y. Zhang, X. Zhang, *Compos. Part B: Eng.* **2023**, *264*, 110916.

[10] Q. He, M. Qin, H. Zhang, J. Yue, L. Peng, G. Liu, Y. Feng, W. Feng, *Mater. Horiz.* **2023**, *11*, 531.

[11] M. Cao, Z. Li, J. Lu, B. Wang, H. Lai, Z. Li, Y. Gao, X. Ming, S. Luo, L. Peng, Z. Xu, S. Liu, Y. Liu, C. Gao, *Adv. Mater.* **2023**, *35*, e2300077.

[12] X. Liu, K. Pang, H. Qin, Y. Liu, Y. Liu, C. Gao, Z. Xu, *ACS Nano* **2022**, *16*, 14703.

[13] Y. Tuersun, W. Lin, X. Huang, W. Qiu, P. Luo, M. Huang, S. Chu, *Carbon* **2022**, *194*, 72.

[14] Z. Yu, S. Wei, J. Guo, *J. Mater. Sci.: Mater. Electron.* **2019**, *30*, 10233.

[15] X. Zhang, S. Zhou, B. Xie, W. Lan, Y. Fan, R. Hu, X. Luo, *Compos. Sci. Technol.* **2021**, *213*, 108922.

[16] N. Lu, X. Zhang, K. Huang, P. Tang, C. Liu, H. Wang, Y. Zeng, *Compos. Sci. Technol.* **2025**, *270*, 111280.

[17] S. Guo, M. Wang, Y. Wang, J. Chen, K. Harr, L. He, Y. Zhang, Y. Zhang, B. Wei, J. Liu, *Small Struct.* **2025**, *6*, 2400652.

[18] X.-H. Li, P. Liu, X. Li, F. An, P. Min, K.-N. Liao, Z.-Z. Yu, *Carbon* **2018**, *140*, 624.

[19] K. Xu, Y. Wang, Z. Zhang, M. Li, R. Yang, Y. Guo, J. Zhang, B. Zhu, Y. Zhou, X. Wang, Y. Qin, L. Li, T. Cai, W. Dai, C. Lin, K. Nishimura, X. Wu, N. Jiang, J. Yu, *Adv. Funct. Mater.* **2025**, *35*, 2505225.

[20] L. Kang, H. Niu, L. Ren, R. Lv, H. Guo, S. Bai, *Chem. Eng. J.* **2023**, *463*, 142402.
